# The sunhine induced placebo effect in Major Depressive Disorder patients exhibits gender differences

**DOI:** 10.1192/j.eurpsy.2023.1037

**Published:** 2023-07-19

**Authors:** J. Gailledreau, B. Gailledreau

**Affiliations:** 971, Cabinet tricolore, Sainte Rose, France

## Abstract

**Introduction:**

Sunshine increases placebo effect in major depressive disorder (MDD) patients (Gailledreau et al., 2015). Kokras et al. (2014) showed that sunshine induces different responses in female than male mice in preclinical models of depression.

**Objectives:**

To determine if the sunshine induced placebo effect exhibits gender differences in human

**Methods:**

Data from 9 double-blind, randomized, placebo-controlled studies of antidepressants conducted by the French GICIPI network were reviewed. MADRS (5) or HAM-D 17 (4) were used as the main efficacy tool. For each patient, variation of scores (Delta MADRS/Delta HAM-D) between two consecutive visits were correlated with the average sunshine index observed at noon between these visits. Sunshine indexes were provided by Météo-France. Correlations were computed with Microsoft Excel.

**Results:**

Sunshine increases placebo effect: however analysis of both genders (n=52) demonstrates no statistically significant (NS) correlation (r²=0.0064). Analysis of the males (n=8) demonstrates NS correlation in cloudy (< 1000 Joules/cm²), variable (1000-2000 Joules/cm²) or sunny (> 2000 Joules/cm²) weather. Although analysis of the females (n=44) demonstrates NS correlation as well for cloudy or variable weather (r²=0.0016), there is a strong correlation between sunshine index and Delta MADRS/Delta HAMD for females exposed to sunny weather: r²=0,315, n=20, p<0.01. This correlation is even stronger in the subpopulation of females aged less than 50 years: r²=0.6398, n=12, p<0.001.

**Image:**

**Figure:**
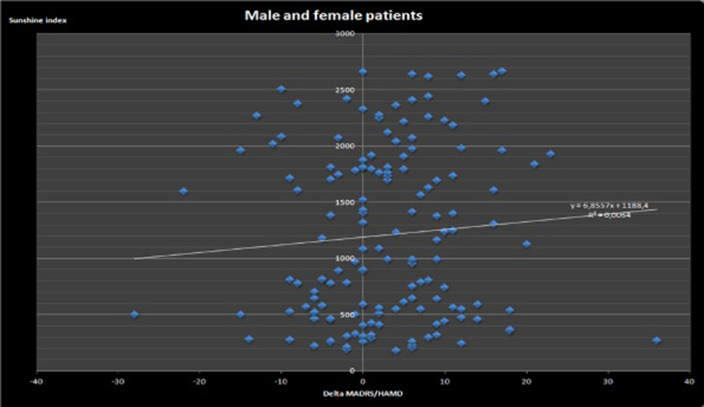

**Image 2:**

**Figure:**
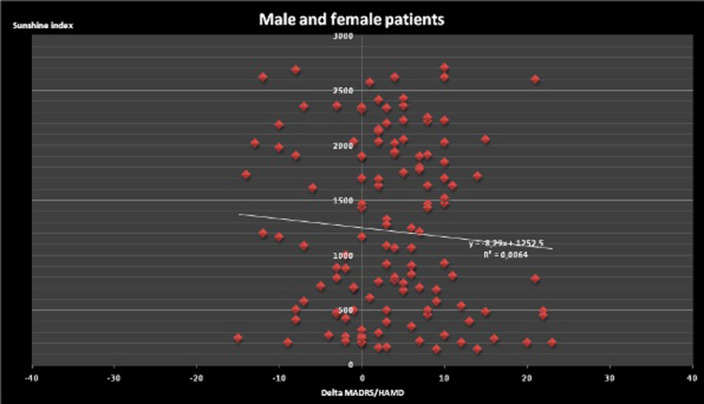

**Image 3:**

**Figure:**
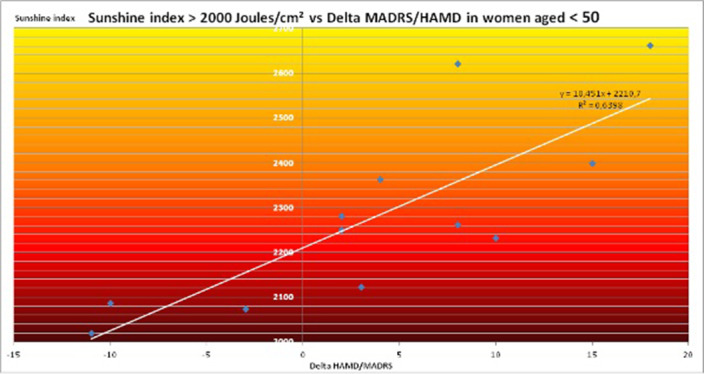

**Conclusions:**

Sunshine increases placebo effect in female patients aged less than 50. This insufficiently known effect may be responsible for failure of a number of double-blind, randomized, studies of antidepressant compounds.

**Disclosure of Interest:**

None Declared

